# The risk of venous thromboembolism and blood hyperlactatemia is associated with increased mortality among critically ill patients with Covid‐19

**DOI:** 10.1111/crj.13628

**Published:** 2023-05-05

**Authors:** Amal F. Alshammary, Hassan M. AlQarni, Raed Farzan, Imran Ali Khan, Vishal Vennu

**Affiliations:** ^1^ Department of Clinical Laboratory Sciences, College of Applied Medical Sciences King Saud University Riyadh Saudi Arabia; ^2^ Clinical Pharmacy Resident King Saud University Medical City Riyadh Saudi Arabia; ^3^ Department of Rehabilitation Sciences, College of Applied Medical Sciences King Saud University Riyadh Saudi Arabia

**Keywords:** blood hyperlactatemia, Covid‐19, creatinine, intensive care unit, lactate, mortality, venous thromboembolism

## Abstract

**Introduction:**

Coronavirus disease 2019 (Covid‐19) following venous thromboembolism (VTE) and blood hyperlactatemia are associated with higher mortality. However, reliable biomarkers for this association remain to be elucidated. This study investigated the associations of VTE risk and blood hyperlactatemia with mortality among critically ill Covid‐19 patients admitted to the intensive care unit (ICU).

**Methods:**

In this single‐centre retrospective study, we included 171 patients aged ≥18 years with confirmed Covid‐19 admitted to the ICU at a tertiary healthcare clinic in the Eastern region of Saudi Arabia between 1 March 2020 and 31 January 2021. Patients were divided into two groups: survivor and non‐survivor. The survivors have been identified as the patients discharged from the ICU alive. The VTE risk was defined using a Padua prediction score (PPS) >4. The blood lactate concentration (BLC) cut‐off value >2 mmol/L was used to determine the blood hyperlactatemia.

**Results:**

Multi‐factor Cox analysis showed that PPS >4 and BLC >2 mmol/L were more likely to be significantly associated with higher odds of ICU mortality in critically ill Covid‐19 patients (hazard ratio [HR] = 2.80, 95% confidence interval [CI] = 1.00–8.08, *p = 0.050*; HR = 3.87, 95% CI = 1.12–13.45, *p* = 0.033, respectively). The Area under the Curve for VTE and blood hyperlactatemia were 0.62 and 0.85, respectively.

**Conclusion:**

VTE risk and blood hyperlactatemia have been associated with a higher mortality risk in critically ill Covid‐19 patients who are hospitalized in the ICU in Saudi Arabia. According to our findings, these people needed more effective VTE prevention strategies based on a personalized assessment of their risk of bleeding. Moreover, persons without diabetes and other groups with a high risk of dying from COVID‐19 may be recognized by measuring glucose as having elevated glucose and lactate jointly.

## Graphical abstract

Critically ill Covid‐19 patients treated in the ICU in Saudi Arabia had a greater mortality risk when they had VTE risk and blood hyperlactatemia. These findings indicated that these individuals required more efficient VTE preventive methods based on evaluating their bleeding risk and monitoring together glucose and lactate, including in people without diabetes.

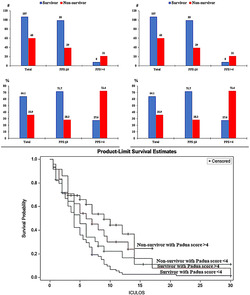

## 1 INTRODUCTION

The clinical scope of coronavirus disease 2019 (Covid‐19) ranges from favourable to severe.^1^ Almost 5% of all symptomatic patients with Covid‐19 are categorized as critically ill, resulting in being moved to the intensive care unit (ICU).^2^ A mortality rate of 49% arises among them due to life‐threatening illnesses.^3^ Several studies have investigated the factors, including laboratory parameters related to a poor ICU outcome and impacting mortality in hospitalized patients with Covid‐19.^4^, ^5^

Few studies have concentrated on the analysis of influencing morbidity and mortality in critically ill patients with Covid‐19.^6^ Only some studies have examined the association between prognostic aspects and mortality risk in these patients, particularly in Saudi Arabia.^7^ A recent study identified cardiovascular, respiratory and renal comorbidities as independent risk factors for Covid‐19 critical outcomes.^8^ Moreover, this study has identified no significant differences in mortality rates among males and females and across different age groups, body mass index (BMI) and nationality. However, other studies have shown that old age, male sex, fever, smoking, comorbidities including diabetes mellitus, asthma, cardiac patients, chronic respiratory diseases, and the presence of two or more comorbidities were significantly associated with Covid‐19 severity.^9^

A recent study^10^ that included Covid‐19 patients and used the Padua prediction score (PPS) to identify in‐hospital venous thromboembolism (VTE) and bleeding risk discovered that critically sick COVID‐19 patients in Shanghai, China had a significant risk of thrombosis and bleeding. Another recent study^11^ indicated that severely ill Covid‐19 patients admitted to the ICU in New York with early‐onset hyperglycemia were most at risk for both 14‐ and 60‐day in‐hospital death. Although several studies have investigated the associations of anticoagulation and blood lactate concentration (BLC) with Covid‐19 mortality risk with varying results in different populations,^7^, ^12^ the results of those studies have been very inconsistent.

Given these few studies that identified various risk factors for mortality among critically ill Covid‐19 patients admitted to ICU in various countries, it remains necessary to investigate the association between the risk of VTE, blood hyperlactatemia and ICU mortality among these patients in Saudi Arabia. Because public awareness about VTE has been reported as poor in Saudi Arabia.^13^ Therefore, this study investigated the association between VTE, blood hyperlactatemia and ICU mortality risk among critically ill patients with Covid‐19 in Saudi Arabia.

## 2 MATERIALS AND METHODS

### 2.1 Study design and setting

This single‐centre retrospective cross‐sectional study examined medical records of confirmed Covid‐19 patients admitted to ICU at tertiary healthcare clinic in the Eastern region of Saudi Arabia between 1 March 2020 and 31 January 2021.

### 2.2 Study population

We included 171 patients aged ≥18 years with Covid‐19 who had not received the Covid‐19 vaccine. The reason to include patients without vaccination was that recent studies^14^, ^15^ indicate an increased risk of VTE due to Covid‐19 vaccination. The infection was diagnosed using the reverse transcription polymerase chain reaction test in throat swabs, sputum or lower respiratory tract samples. These contained patients were categorized into two groups: survivor and non‐survivor.^16^ The patients discharged from the ICU alive have been identified as survivors. On the other hand, those whose ICU discharge disposition was death have been classified as non‐survivors. Patients were excluded if they had bleeding before ICU admission.

### 2.3 Exposure and outcome variables

The PPS was created to determine the likelihood of VTE risk in hospitalized patients. A PPS of ≤4 represents a low risk of VTE. This is one of the acknowledged scoring techniques suggested for evaluating the risk of VTE in non‐surgical patients both during and after hospitalization.^17^ According to another study, the cumulative PPS can effectively stratify the risk of VTE in these inpatients.^18^ The event of lower limb deep vein thrombosis (DVT), pulmonary embolism (PE)^19^ or both, which were suspected by the treatment team and identified by Doppler ultrasonography (for DVT) or CT pulmonary angiography (for PE),^16^ are all examples of clinically suspected and confirmed VTE. Blood hyperlactatemia was determined as a BLC cut‐off value >2 mmol/L.^20^, ^21^ All these parameters were acquired by reviewing patients' medical files. The outcome of interest was ICU mortality.

### 2.4 Covariates

For this study, the following several covariates were collected from critically ill patients with Covid‐19 at admission to ICU who had not received the Covid‐19 vaccine: demographics (age, gender), and clinical signs, such as temperature, systolic and diastolic blood pressure, the fraction of inspired oxygen (FiO2), presence of comorbidities, Sequential Organ Failure Assessment (SOFA) and Acute Physiology and Chronic Health Evaluation (APACHE) III scores, weight loss, last body mass index (BMI), heart rate (HR), respiratory rate (RR), an anticoagulant used, and ventilator use.

### 2.5 Statistical analysis

The constant variables were briefed in mean and standard deviation (SD) or median and interquartile range (IQR) based on the data distribution for survivors and non‐survivors. The categorical variables were displayed in count and percentages. Significance between the groups was defined using the independent Student's *t*‐test, a one‐way analysis of variance for continuous variables. In contrast, the Mann–Whitney, Kruskal–Wallis or Chi‐square tests were utilized for definite variables. The clinical features and laboratory test results, including normal ranges, were shown in count and median (IQR).

Cox regression was used to evaluate the association between VTE risk, blood hyperlactatemia and ICU mortality. The association was estimated in two steps: univariate and multivariate. Multivariate model adjusting for age, gender, creatinine clearance, creatinine, platelet count, ferritin level, and baseline D‐dimer levels were all adjusted for in a multivariate model. The data were reported as hazard ratio (HR) and 95% confidence interval (CI). Survivor was used as a reference. The area under the curve was generated to identify predictors of mortality based on the highest sensitivity and specificity in four groups: (1) non‐survivor with VTE, (2) non‐survivor without VTE, (3) survivor with VTE and (4) survivor without VTE. The survival curve was drawn using the Kaplan–Meier method. The variable group represents the patient's risk category, the ICULOS represents the ICU stay, and the variable Status is the censoring indicator. The log‐rank test established the significance of survival trends in various groups. All the analyses were conducted using SAS version 9.4 (SAS corporation, NC, USA) for Windows. The statistical significance was determined as *p* < 0.05.

## 3 RESULTS

**FIGURE 1:**
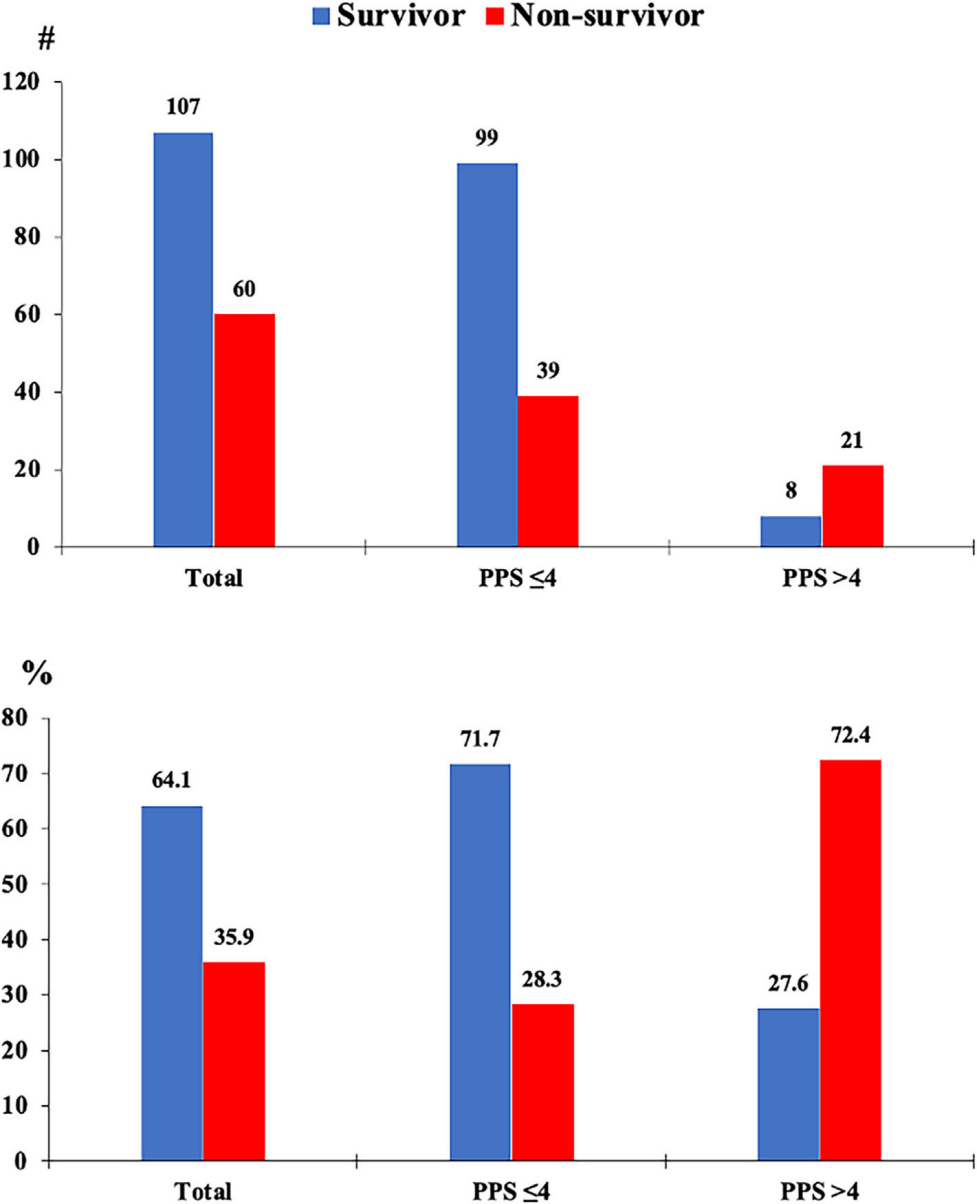
Padua prediction score frequency (up) and proportion (down) for survivors and non‐survivors.

**FIGURE 2:**
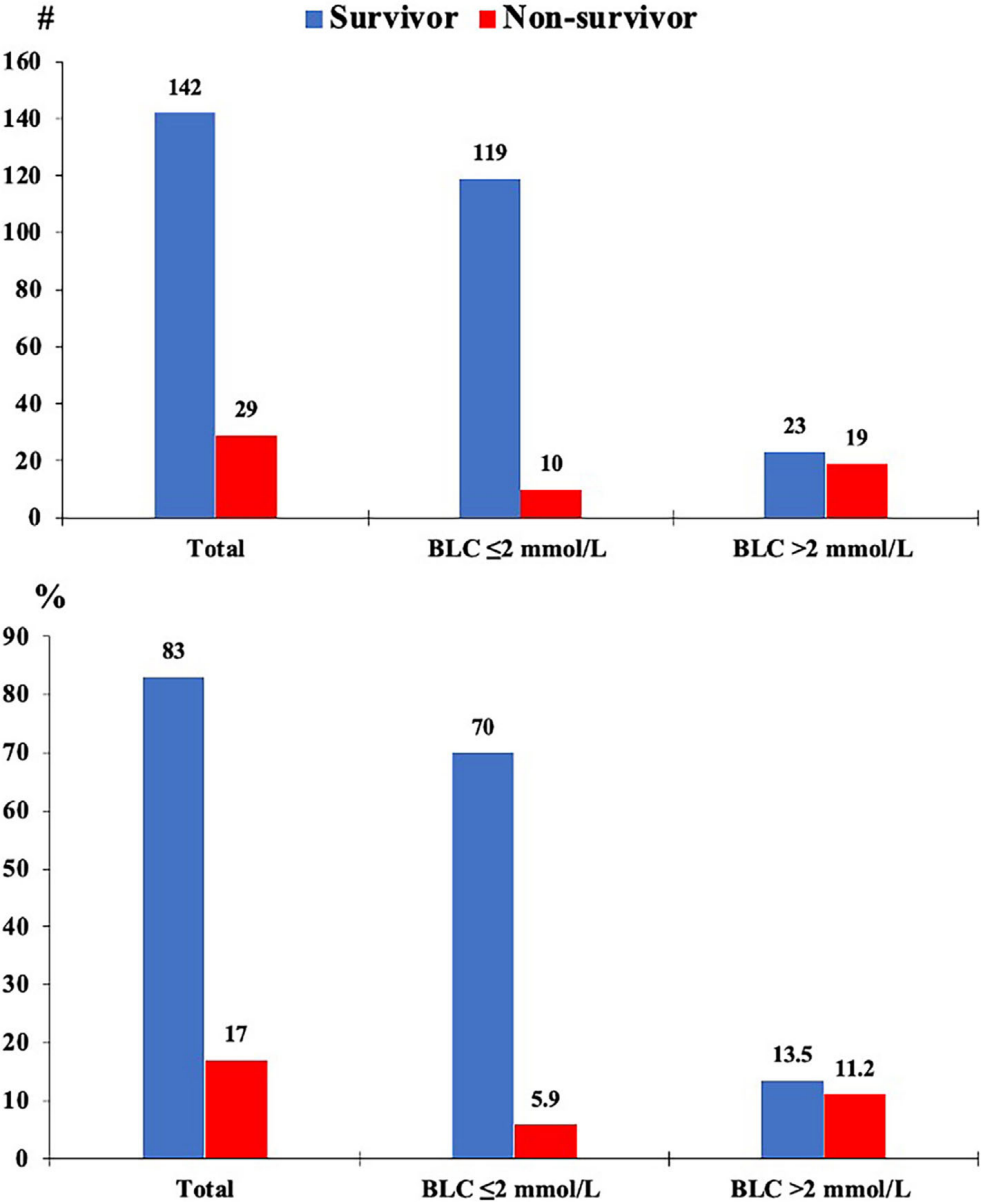
Blood lactate concentration score frequency (up) and proportion (down) for survivors and non‐survivors.

Of the 171 patients, 83% were survivors and 27% were non‐survivors. Although the average age of the total patients was 65.9 years, non‐survivors (58.9 years old) were 14 years younger than the survivors (73 years old). Most non‐survivors had a higher median score for the SOFAS (3) and FO2 (32). Moreover, non‐survivors had less creatinine clearance (median: 26.2 mL/min) and higher creatinine (mean: 2.4 mg/dL). Non‐survivors had lost platelets (mean: 278.1 cells/mm3), including those with high lactic acid (median: 3 mmol/L) and iron (mean: 85.3 g/dL). In addition, non‐survivors had higher ferritin levels (median: 1079 mg/L). The median length of hospital and ICU LOS for these patients was 18 and 8 days, respectively (Table 1). The proportion of PPS >4 was significantly high in non‐survivors (72.4%) (Figure 1). The proportion of BLC >2 mmol/L was 11.2% in non‐survivors (Figure 2).

**TABLE 1:** Characteristics of the sample, stratified into survivor and non‐survivor.

	Total *n* = 171	Survivor *n* = 142 (83%)	Non‐survivor *n* = 29 (17%)	*p*‐value
Age in year, mean (SD)	65.9 (11.8)	73 (10.6)	58.9 (13.1)	˂0.0001
Age group, *n* (%)				˂0.0001
˂65 years	105 (61.4)	99 (69.7)	6 (20.7)	
≥ 65 years	66 (38.6)	43 (30.3)	23 (79.3)	
Sex, *n* (%)				0.944
Males	113 (66.1)	94 (66.2)	19 (66.5)	
Females	58 (33.9)	48 (33.8)	10 (34.5)	
The anticoagulant used, *n* (%)				0.3719
Heparin	16 (9.5)	12 (8.6)	4 (13.8)	
Enoxaparin	133 (78.7)	113 (80.7)	20 (69)	
Both/rivaroxaban	20 (11.8)	15 (10.7)	5 (17.2)	
Anticoagulant dose, *n* (%)				0.810
Prophylactic	56 (32.9)	47 (33.3)	9 (31)	
Therapeutic	114 (67.1)	94 (66.7)	20 (69)	
Respiratory rate (cycles/min), median (IRQ)	21 (15–22)	18 (15–20)	20 (15–25)	
Heart rate (beats/min), mean (SD)	87.1 (13.8)	83.1 (13.4)	91.1 (14.3)	
Systolic BP (mm Hg), mean (SD)	127.7 (20.1)	126.3 (18.2)	129.2 (22.1)	0.447
Systolic BP (mm Hg), mean (SD)	73.5 (10.8)	74.1 (10.9)	73.0 (10.8)	0.612
SOFA score, median (IRQ)	3 (2–9)	2 (2–11)	3 (2–7)	˂0.0001
Padua prediction score, n (%)				˂0.0001
4 or less	107 (64.1)	99 (71.7)	8 (27.6)	
More than 4	60 (35.9)	39 (28.3)	21 (72.4)	
FO2, median (IRQ)	30 (10.5–34)	28 (0–32)	32 (21–36)	˂0.0001
Temperature (°C), median (IRQ)	37 (36–37)	37 (36–37)	37 (36–37)	˂0.0001
Ventilator used, *n* (%)				˂0.0001
No	148 (87.6)	133 (95)	15 (51.7)	
Yes	21 (12.4)	7 (5)	14 (48.3)	
Comorbidities, *n* (%)				0.282
0 or 1	43 (25.2)	38 (26.8)	5 (17.2)	
≥2	128 (74.8)	104 (73.2)	24 (82.8)	
Creatinine clearance, median (IRQ)	54.4 (7.2–72.3)	82.6 (6.2–105.6)	26.2 (8.8–39.1)	˂0.0001
Creatinine, mean (SD)	1.7 (1.6)	1.1 (1.5)	2.4 (1.8)	0.001
Last platelets, mean (SD)	226.5 (109.1)	174.9 (114.1)	278.1 (104.2)	˂0.0001
Last lactic acid, median (IRQ)	2.2 (0.4–3.2)	1.4 (0.7–2)	3 (0.9–4.5)	˂0.0001
Last iron, mean (SD)	71.4 (37.9)	57.6 (27.5)	85.3 (48.4)	0.045
Ferritin level, median (IRQ)	807 (28–1620)	535 (10–1098)	1079 (46–2143)	˂0.0001
Baseline D‐dimer, median (IRQ)	0.7 (0–1.1)	0.5 (0–1)	0.9 (0–1.3)	˂0.0001
Hospital length of stay, median (IRQ)	15 (4–20)	12 (2–19)	18 (6–21)	˂0.0001
Length of stay in ICU, median (IRQ)	6 (0.7–10)	4 (0.5–7)	8 (1–13)	˂0.0001

Abbreviations: BP, blood pressure; ICU, intensive care unit; IRQ, interquartile range; SD, standard deviation.

The PPS >4 was substantially associated with higher odds of ICU mortality in non‐survivors than survivors (HR = 6.66, 95% CI = 2.72–16.30, *p* < 0.0001). The BLC >2 mmol/L was also significantly associated with greater odds of ICU mortality in this group (HR = 1.40, 95% CI = 1.10–1.79, *p* < 0.0001; HR = 2.30, 95% CI = 1.54–3.42; *p* < 0.0001, respectively). The multivariate model adjusted for age, gender, creatinine clearance, creatinine, platelets, ferritin level and baseline D‐dimer revealed that PPS >4 and BLC >2 mmol/L were associated with higher odds of ICU mortality (HR = 2.80, 95% CI = 1.00–8.08, *p* = 0.050; HR = 3.87, 95% CI = 1.12–13.45; *p* = 0.033, respectively) (Table 2).

**TABLE 2:** Univariate and multivariate models for mortality in the intensive care unit.

	Univariate analysis		Multivariate analysis	
	HR (95% CI)	*p*‐Value	HR (95% CI)	*p*‐Value
PPS >4	6.66 (2.72—16.30)	˂0.0001	2.80 (1.00–8.08)	0.050
BLC >2 mmol/L	2.30 (1.54—3.42)	˂0.0001	3.87 (1.12–13.45)	0.033

Abbreviations: ICU, intensive care unit; HR, hazard ratio, CI, confidence interval; BLC, blood lactate concentration; PPS, Padua prediction score.

**FIGURE 3:**
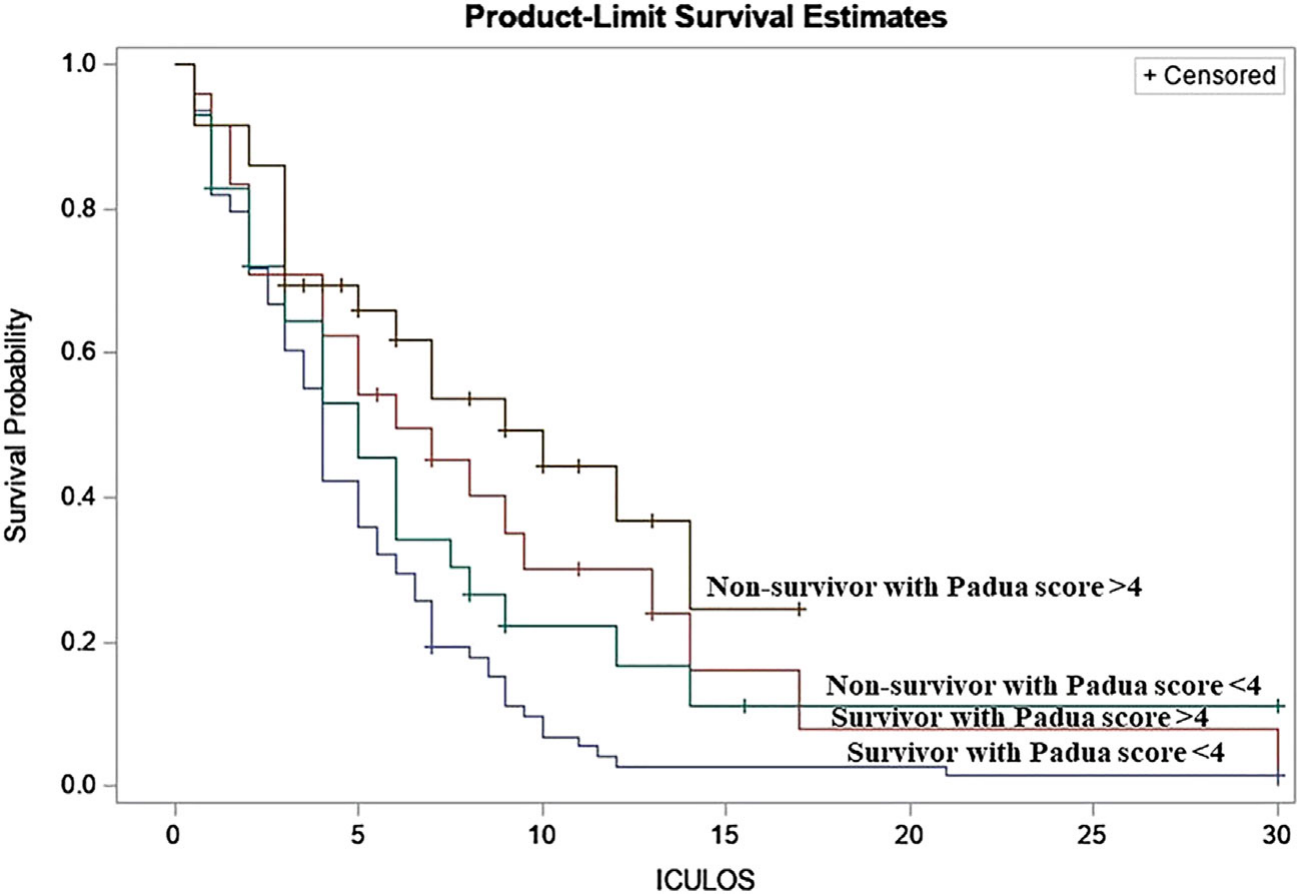
Survival probability in intensive care according to Padua prediction score among survivors and non‐survivors.

The area under curve (AUC) for PPS >4 and BLC >2 mmol/L were 0.62 and 0.85, respectively (Table 3). There was a significant difference in ICU LOS between the groups (log‐rank test, *p* = 0.0004). At 18 days of ICU LOS, the survival probability estimates were about 0.3 for non‐survivors with PPS >4. For the other groups, the probability of survival after 30 days of ICU LOS was approximately 0.1 (Figure 3).

**TABLE 3:** The results about the area under the curve for ICU mortality.

	AUC	95% CI	*p*‐Value
PPS >4	0.620	0.477–0.764	0.422
BLC >2 mmol/L	0.854	0.705–1.00	0.351

Abbreviations: AUC, the area under the curve; BLC, blood lactate concentration; CI, confidence interval; PPS, Padua prediction score.

## 4 DISCUSSION

The present study investigated the association between VTE risk, blood hyperlactatemia and ICU mortality risk among critically ill Covid‐19 patients in Saudi Arabia. The results of this study demonstrated that VTE risk and blood hyperlactatemia were significantly associated with greater odds of ICU mortality among non‐survivors than survivors of Covid‐19 who had not received the Covid‐19 vaccine, even after adjusting for age, gender, creatinine clearance, creatinine, platelets, ferritin level and baseline D‐dimer.

The AUC of blood hyperlactatemia was close to 0.8, indicating that the predictive abilities of mortality risk were acceptable.^22^ However, the AUC of VTE was close to 0.6, indicating that the predictive ability of mortality risk was close to poor or a toss of a coin. Moreover, the Kaplan–Meier survival curve illustrates a significant difference in ICU LOS survival between the groups.

The current study found that 72.4% of Covid‐19 non‐survivor patients experienced VTE risk in the ICU, which was high compared to the prevalence reported in the previous studies.^23^, ^24^ Blood hyperlactatemia occurred in 11.2% of non‐survivor patients during the ICU LOS, less than in an earlier study.^25^ Just 28.3% and 5.9% of non‐survivors, respectively, had neither a VTE risk nor blood hyperlactatemia.

In the present study, the finding from the multivariate analysis shows that VTE significantly had 80% more likely associated with mortality risk among non‐survivors than among survivors in Saudi Arabia. This finding is consistent with an earlier meta‐analysis showing that VTE incidence was 31% among critically ill Covid‐19 patients, followed by DVT, PE and arterial thromboembolism, 28%, 19% and 5%, respectively.^26^ That study also concluded that critically ill Covid‐19 patients are at risk of thrombosis (both arterial and venous), despite heparin treatment, especially because it has been reported that older age is a potential risk factor for a more severe disease course, including in Saudi Arabia.^27^ Another recent meta‐analysis found that the incidence of VTE among hospitalized Covid‐19 patients was high and was associated with an increased risk of mortality in these patients, particularly among patients in ICU.^12^ A possible explanation for this might be that the mechanism of Covid‐19 thrombosis does not clear yet.^26^ Another explanation is that many patients are probably not being screened by computed tomography, pulmonary angiography, or compression ultrasonography or that many thrombotic events may be underestimated due to the severe condition of Covid‐19 patients brought to the ICU.^28^ Microvascular thromboses are difficult to evaluate and often impossible to differentiate from other causes of organ dysfunction without autopsies.^29^

Consistent with the literature,^20^ this research also found that blood hyperlactatemia has been significantly associated with high mortality risk among critically ill Covid‐19 patients in Saudi Arabia. A recent systematic literature review explored the possible association between increased blood lactate levels and mortality in Covid‐19 patients and compared lactate values between Covid‐19 and non‐Covid‐19 patients.^7^ It was found that higher lactate values tend to display a worse outcome in Covid‐19 patients. However, these study patients did not have sustained baseline hyperlactatemia, and substantially elevated lactate values were not consistently present in patients with worse clinical outcomes. Overall, findings from this study suggested that blood lactate monitoring upon admission and throughout hospitalization might be useful for the early identification of worse clinical outcomes in Covid‐19 patients. Previous study have demonstrated that lactic acid and creatinine were high among non‐survivor patients.^30^ This result indicated that high lactate and creatinine levels are closely related to the poor prognosis of patients. This result may be explained by the fact that the rising biomarkers, especially lactate and creatinine levels, can be used as indicators of disease progression.

The primary strength of this study is that it first investigated the association between risk in VTE, blood hyperlactatemia and ICU mortality risk among critically ill Covid‐19 patients in Saudi Arabia using a PPP >4 and BLC >2 mmol/L as biomarkers. Some studies found PPP >4 to be well in populations of Italy^17^ and China.^18^ Surprisingly, this study's results indicated that the post‐ICU admission mortality rate was 17%, which is relatively lower than initial reports among patients from China, Europe and the United States.^27^ This study has some limitations. The results from this study need to be interpreted with caution mainly due to its retrospective, single‐centre design. In particular, it was limited to some laboratory test results, and detailed imaging data were unavailable. Unfortunately, not all patients will have access to this imaging data because of the retrospective nature of this study. It limits the validity of this study's findings.

## 5 CONCLUSION

VTE risk and blood hyperlactatemia were linked to a high mortality risk among critically ill patients with Covid‐19 who were hospitalized in the ICU. The single‐centre retrospective methodology and our study's small sample size were both limitations. Our findings, however, imply that more successful VTE prevention methods based on an individual evaluation of the risk of bleeding were required for these individuals. Additionally, a group with a high chance of dying from COVID‐19, including those without diabetes, may be identified by measuring glucose as having increased glucose and lactate together. More research is required to examine all potential factors implicated in mortality. Clinical trials are necessary to ascertain if preventive or therapeutic anticoagulation reduces the mortality risk.

## AUTHOR CONTRIBUTIONS

*Conceptualization*: Hassan M. AlQarni and Vishal Vennu. *Data curation*: Hassan M. AlQarni and Vishal Vennu. *Formal analysis*: Vishal Vennu. *Funding acquisition*: Imran Ali Khan. *Investigation*: Vishal Vennu. *Methodology*: Hassan M. AlQarni and Amal F. Alshammary. *Project administration*: Hassan M. AlQarni. *Resources*: Hassan M. AlQarni. *Software*: Vishal Vennu. *Supervision*: Vishal Vennu. *Validation*: Amal F. Alshammary, Raed Farzan and Imran Ali Khan. *Visualization*: Amal F. Alshammary, Raed Farzan and Imran Ali Khan. *Writing—original draft*: Vishal Vennu and Hassan M. AlQarni. *Writing—review and editing*: Amal F. Alshammary, Raed Farzan and Imran Ali Khan. *Approval of final manuscript*: Amal F. Alshammary, Hassan M. AlQarni, Raed Farzan, Imran Ali Khan and Vishal Vennu.

## ACKNOWLEDGMENTS

The authors would like to extend their sincere appreciation to the Researchers Supporting Project number (RSPD2023R735), King Saud University, Riyadh, Saudi Arabia for funding this project.

## CONFLICT OF INTEREST STATEMENT

There are no conflicts of interest.

## ETHICS STATEMENT

This study was performed in line with the principles of the Declaration of Helsinki. This study was approved by the National Committee of the Bio‐Ethics at John Hopkins Aramco Healthcare (reference #H‐05‐DH‐044) on 7 April 2021. Written consent was obtained from the patients before enrolling in this study. All methods were carried out following the necessary laws and regulations.

## Data Availability

The data supporting this study's findings are available from the corresponding author upon reasonable request.
